# Retinal mid-peripheral capillary free zones are enlarged in cognitively unimpaired older adults at high risk for Alzheimer’s disease

**DOI:** 10.1186/s13195-023-01312-8

**Published:** 2023-10-12

**Authors:** Edmund Arthur, Swetha Ravichandran, Peter J. Snyder, Jessica Alber, Jennifer Strenger, Ava K. Bittner, Rima Khankan, Stephanie L. Adams, Nicole M. Putnam, Karin R. Lypka, Juan A. Piantino, Stuart Sinoff

**Affiliations:** 1 School of Optometry, University of Alabama at Birmingham, Birmingham, AL USA; 2 Department of Neurology, Alpert Medical School of Brown University, Providence, RI USA; 3 Department of Biomedical and Pharmaceutical Sciences, University of Rhode Island, Kingston, RI USA; 4 George and Anne Ryan Institute for Neuroscience, University of Rhode Island, Kingston, RI USA; 5 Butler Hospital Memory & Aging Program, Providence, RI USA; 6 Department of Psychiatry and Human Behavior, Alpert Medical School of Brown University, Providence, RI USA; 7 Department of Ophthalmology, Stein Eye Institute, University of California Los Angeles, Los Angeles, CA USA; 8 Southern California College of Optometry, Marshall B. Ketchum University, Fullerton, CA USA; 9 Illinois College of Optometry, Chicago, IL USA; 10 State University of New York College of Optometry, New York, NY USA; 11 Bascom Palmer Eye Institute, University of Miami, Miami, FL USA; 12 Department of Pediatrics, Oregon Health & Science University, Portland, OR USA; 13 Providence-Swedish Health System, Seattle, WA USA

**Keywords:** Alzheimer’s disease, Periarteriole capillary free zones, Perivenule capillary free zones, Mid-peripheral capillary free zones, Early risk detection, APOE genotyping, Optical coherence tomography angiography

## Abstract

**Background:**

Compared to standard neuro-diagnostic techniques, retinal biomarkers provide a probable low-cost and non-invasive alternative for early Alzheimer’s disease (AD) risk screening. We have previously quantified the periarteriole and perivenule capillary free zones (mid-peripheral CFZs) in cognitively unimpaired (CU) young and older adults as novel metrics of retinal tissue oxygenation. There is a breakdown of the inner retinal blood barrier, pericyte loss, and capillary non-perfusion or dropout in AD leading to potential enlargement of the mid-peripheral CFZs. We hypothesized the mid-peripheral CFZs will be enlarged in CU older adults at high risk for AD compared to low-risk individuals.

**Methods:**

20 × 20° optical coherence tomography angiography images consisting of 512 b-scans, 512 A-scans per b-scan, 12-µm spacing between b-scans, and 5 frames averaged per each b-scan location of the central fovea and of paired major arterioles and venules with their surrounding capillaries inferior to the fovea of 57 eyes of 37 CU low-risk (mean age: 66 years) and 50 eyes of 38 CU high-risk older adults (mean age: 64 years; *p* = 0.24) were involved in this study. High-risk participants were defined as having at least one APOE e4 allele and a positive first-degree family history of AD while low-risk participants had neither of the two criteria. All participants had Montreal Cognitive Assessment scores ≥ 26. The mid-peripheral CFZs were computed in MATLAB and compared between the two groups.

**Results:**

The periarteriole CFZ of the high-risk group (75.8 ± 9.19 µm) was significantly larger than that of the low-risk group (71.3 ± 7.07 µm), *p* = 0.005, Cohen’s *d* = 0.55. The perivenule CFZ of the high-risk group (60.4 ± 8.55 µm) was also significantly larger than that of the low-risk group (57.3 ± 6.40 µm), *p* = 0.034, Cohen’s *d* = 0.42. There were no significant differences in foveal avascular zone (FAZ) size, FAZ effective diameter, and vessel density between the two groups, all *p* > 0.05.

**Conclusions:**

Our results show larger mid-peripheral CFZs in CU older adults at high risk for AD, with the potential for the periarteriole CFZ to serve as a novel retinal vascular biomarker for early AD risk detection.

## Background

Globally, individuals with Alzheimer’s disease (AD) dementia, mild cognitive impairment (MCI) due to AD, and preclinical AD are estimated at 32, 69, and 315 million, respectively. Altogether, these constitute 416 million people across the AD continuum or 22% of all persons aged 50 and above [1]. AD is the most common cause of dementia in the elderly, and a progressive neurodegenerative disease affecting approximately 6.7 million Americans aged 65 and older, and projected to reach 13.8 million by 2060 [2]. It is in the top 10 leading causes of death in America and has no proven preventative or curative interventions [3]. Early diagnostics are critical for the development of effective therapies. The pathophysiological process of AD occurs decades before symptoms of dementia emerge [4–9]. It is therefore critical to develop non-invasive/cost-efficient biomarkers to aid in the early detection and interventions to prevent or delay dementia onset. While positron emission tomography (PET) and cerebrospinal fluid (CSF) assessment via lumbar puncture have the greatest utility, they are not routinely used because they are expensive and invasive. While rapid advances in blood-based biomarkers will likely become part of the normal clinical diagnostic pathway within the next few years [10–13], there is still the need for other non-invasive biomarkers.

There is increasing evidence that there are retinal manifestations of AD; the foveal avascular zone (FAZ) area is enlarged; retinal vessel density, central macular, and choroidal thickness are reduced in individuals with a genetic risk (apolipoprotein E; APOE e4) and first-degree family history of AD [14–17]. The eye and brain are anatomically, embryologically, and physiologically linked. The retinal ganglion cells (RGCs) are similar to the cerebral cortex neurons, and the cerebral small vessels are similar to retinal vessels [18, 19]. The human retina is an easily accessible part of the central nervous system (CNS) and an ideal target for the identification of AD risk biomarkers.

The FAZ and vessel density areas proposed as retinal vascular biomarkers for early AD detection all have limitations that decrease their effect size or clinical relevance for early disease detection [20–24]. One issue with using the FAZ area as a biomarker for AD is that it is limited to a few or a single layer of capillaries. Moreover, it may become saturated with increasing disease severity and not increase further in size, even with the loss of capillaries [22, 23]. FAZ area is highly variable in cognitively unimpaired (CU) older adults [23, 25–28], and studies have shown that the FAZ area is not consistently significantly larger in an AD cohort than a control cohort [29–32]. Also, vessel density measurements in AD patients using optical coherence tomography angiography (OCTA) [29, 33–35] are influenced by noise in the image, along with variable anatomic features such as vessel diameter [20, 21, 23].

In previous studies, we characterized and quantified the metrics of tissue oxygenation in the retina of young and older CU participants in the form of periarteriole and perivenule capillary free zones (mid-peripheral CFZs) [22, 23]. The mid-peripheral CFZs represent the maximum distance that oxygen and nutrients must diffuse to reach the retinal neurons with larger distances indicating potential ischemia [22, 23]. There is a breakdown of the inner retinal blood barrier, pericyte loss, and capillary non-perfusion or dropout in AD [36, 37] leading to potential enlargement of the mid-peripheral CFZs around the arterioles and venules in the retina of AD patients.

The goal of the current study was twofold: (1) determine if the mid-peripheral CFZ is a more robust biomarker for early AD risk detection compared to FAZ and vessel density measurements and (2) assess whether a model of combined metrics for mid-peripheral CFZ, FAZ, and vessel density will better distinguish between low-risk and high-risk CU older adults for AD compared to a model of the mid-peripheral CFZs alone. Thus, this proof-of-concept study explored a novel non-invasive, inexpensive retinal vascular biomarker and a model of retinal vascular metrics for their potential to assist with early AD risk detection and disease monitoring. Such multimodal measures of retinal abnormalities may facilitate large-scale screening of older adults and referral of at-risk individuals by point-of-care clinicians to neurologists and neuropsychologists for detailed cognitive health/biomarker assessment.

## Methods

### Study participants

Fifty-seven (57) eyes of 37 CU low-risk (age; mean: 66 years; range: 56–80 years; 18 males and 19 females) and 50 eyes of 38 CU high-risk older adults (age; mean: 64 years; range: 55–77 years; 11 males and 27 females) were involved in this current study (total of 107 eyes in 75 participants). There were no significant differences in age; *t*(73) = 1.19, *p* = 0.24 or proportion with respect to sex; *X*^2^ (1, *N* = 75) = 3.07, *p* = 0.08 between the two groups. All participants had refractive errors of ≤  ± 5.00 DS (spherical equivalent; equivalent axial length of ~ 21–26 mm) to prevent significant differences in retinal magnification in the OCTA images as noted by the Bennett formula [38]. Also, all CU participants had Montreal Cognitive Assessment (MoCA) scores of ≥ 26 [39, 40] and Repeatable Battery for the Assessment of Neuropsychological Status Update (RBANS-U) Delayed Memory Index (DMI) scores of ≥ 85 [41, 42]. CU high-risk participants were defined as individuals with at least one allele of the APOE e4 gene and a first-degree family history of AD while CU low-risk participants were non-carriers for the APOE e4 allele and no first-degree family history of AD. The inclusion criteria for participants in the study were as follows: absence of or controlled hypertension (< 140/90) and hyperlipidemia and no systemic diabetes (HbA1c ≤ 7). The exclusion criteria for our study were as follows: unstable doses of antidepressants that have significant anticholinergic side effects; age-related macular degeneration; diabetic retinopathy; glaucoma; retinal ischemic conditions; large cataracts that will impede imaging; current intake of retino toxic drugs such as chloroquine, hydroxychloroquine, and cancer drugs; and other neurodegenerative diseases such as Parkinson’s disease or multiple sclerosis. Smoking history has previously been shown to have a significant effect on retinal vasculature [43]. There was no significant difference in the total years of smoking between the two groups, *p* = 0.22. All participants had best corrected visual acuity of ≥ 20/40 (~ LogMAR 0.30). An estimated sample size (*N* = 72) was computed with a GPower 3.1 calculator [44] using the following input parameters from a previous study in preclinical AD [30]: effect size (CU low risk vs. CU high risk; Cohen’s *d*) of 0.60, *α* of 0.05, and power of 0.80. Considering 107 eyes of 75 participants were involved in this study, our study was adequately powered to detect the differences in the OCTA parameters. The study adhered to the tenets of the Declaration of Helsinki, and informed consent from all subjects was obtained prior to the experimental data collection after the explanation of the nature and possible consequences of the study. The study was part of the Atlas of Retinal Imaging in Alzheimer’s Study (ARIAS; PJS served as principal investigator for ARIAS) which took place at the University of Rhode Island and Butler Hospital Memory and Aging Program, Providence, RI, between 2020 and 2022 and was approved by the BayCare Institutional Review Board (IRB).

### Neuropsychological evaluation with MoCA and RBANS-U

All study participants underwent detailed neuropsychological evaluation with MoCA [39, 40] and RBANS-U [41, 42]. The MoCA test [39, 40] is a one-page, 30-point test administered in approximately 10 min. A score of 26 or over is considered CU. It assesses several cognitive domains including short-term memory, visuospatial ability, executive function, attention, concentration and working memory, language, and orientation to time and place. The total MoCA score was assessed for the participants.

The RBANS-U [41, 42] is a brief neuropsychological assessment battery that can be administered to adult patients aged 20–89 years old. The RBANS consists of 10 subtests, which give five scores (one for each of the five domains tested), including immediate memory (immediate list learning and immediate story), visuoperceptual abilities (figure copy and line orientation), language (naming and semantic fluency), attention (digit span forward and digit-symbol coding), and delayed memory (delayed list memory with recognition, delayed story memory, and delayed figure memory). It takes about 30 min to administer. The RBANS-U DMI scores were assessed for our study participants.

### APOE genotyping via cheek swab

DNA was taken from the buccal samples of epithelial tissues collected from the inside of the cheek for each study participant. For reproducibility purposes, a total of 2 swabs were collected from each study participant. The sample was placed into a reagent tube, and both samples were inserted into the DNA analyzer cube (Spartan Bioscience Inc., Montreal, Canada). The test system integrated and automated DNA extraction using PCR-based amplification of the APOE target gene. The system had integrated controls for monitoring run performance and automatically informed the operator of any anomalies in the instrument or reagent. For the purpose of the study, samples were analyzed in real time, and the results were available to the investigator in less than 1-h. Low-risk participants had no allele of the APOE e4 gene (i.e., participants had e2 or e3 gene). High-risk participants had at least one allele of the APOE e4 gene (i.e., e2/e4, e3/e4, or e4/e4).

### Image acquisition with OCTA

**Fig. 1:**
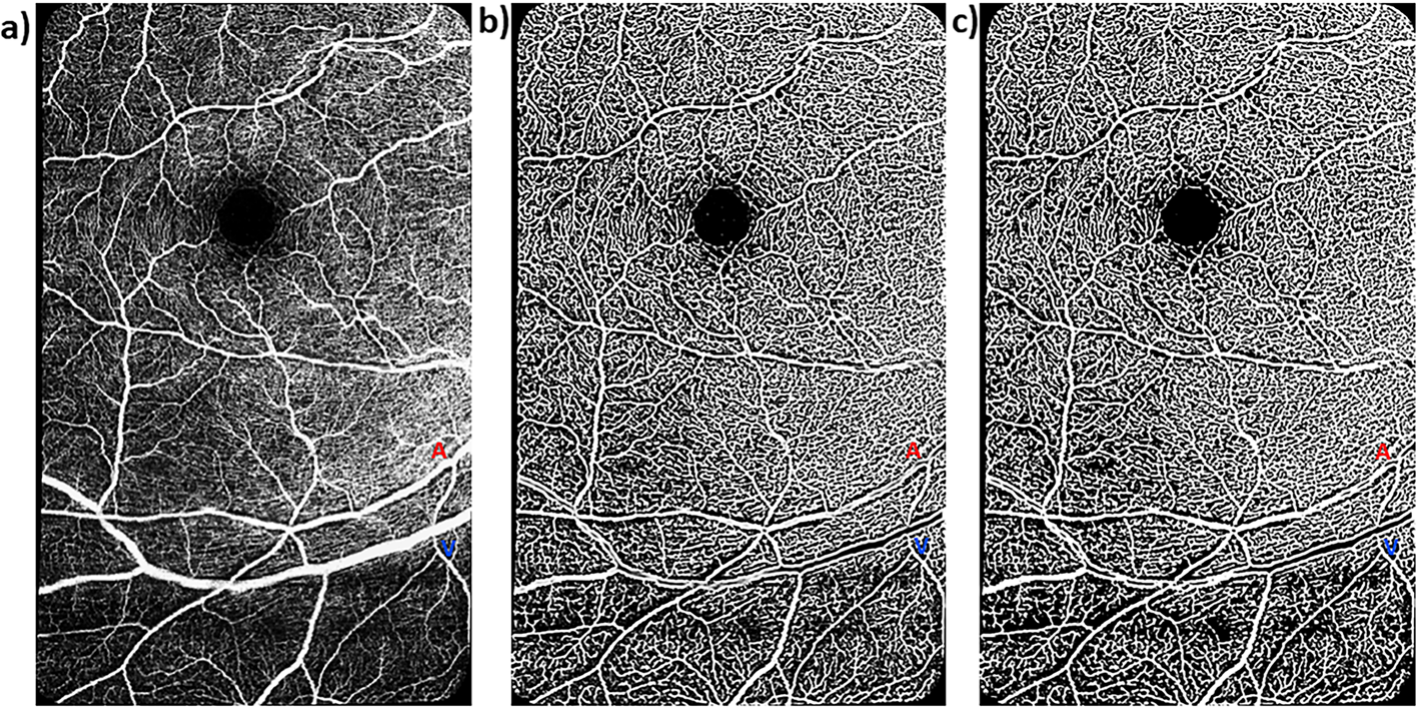
Montaged 20 × 20°OCTA images of the central foveal region and paired arterioles and venules in the superficial vascular plexus of a 60-year-old cognitively unimpaired low-risk female. **a** Raw montaged OCTA image before image processing. **b** Vesselness filtered image in MATLAB. **c** Vesselness filtered and Otsu thresholded image in MATLAB. Red “A” and blue “V” represent paired arterioles and venules, respectively. Periarteriole CFZ can be seen as dark gaps around the arteriole (red “A”) while the perivenule CFZ can be seen as dark gaps around the venule (blue “V”). Periarteriole CFZ can be seen as larger than the perivenule CFZ

Prior to imaging, all participants were dilated with two drops of tropicamide (Mydriacyl 1%) per eye. There was a 15-min wait time from the administration of the dilation drops to image acquisition. All imaging procedures were completed for both the right eye and the left eye. We obtained 20 × 20°OCTA images consisting of 512 b-scans, 512 A-scans per b-scan, 12 µm spacing between the b-scans, and 5 frames averaged per each b-scan location of the central fovea and of paired major arterioles and venules with their surrounding capillaries inferior to the fovea (Spectralis HRA + OCT; Eye Explorer version 1.10.4.0; Heidelberg Engineering, Germany; Fig. 1) as done previously [22, 23]. The signal quality values (range for Spectralis = 0–40) of all our OCTA images from the vendor software were at least 20 to ensure good image quality. In addition, all scans were visually inspected for motion and shadowing artifacts. To increase our sample size, both eyes from participants were selected for the purpose of image analysis so far as they had good image quality. In cases, where only one eye had good image signal quality, only that eye was selected.

### Processing of OCTA images

**Fig. 2:**
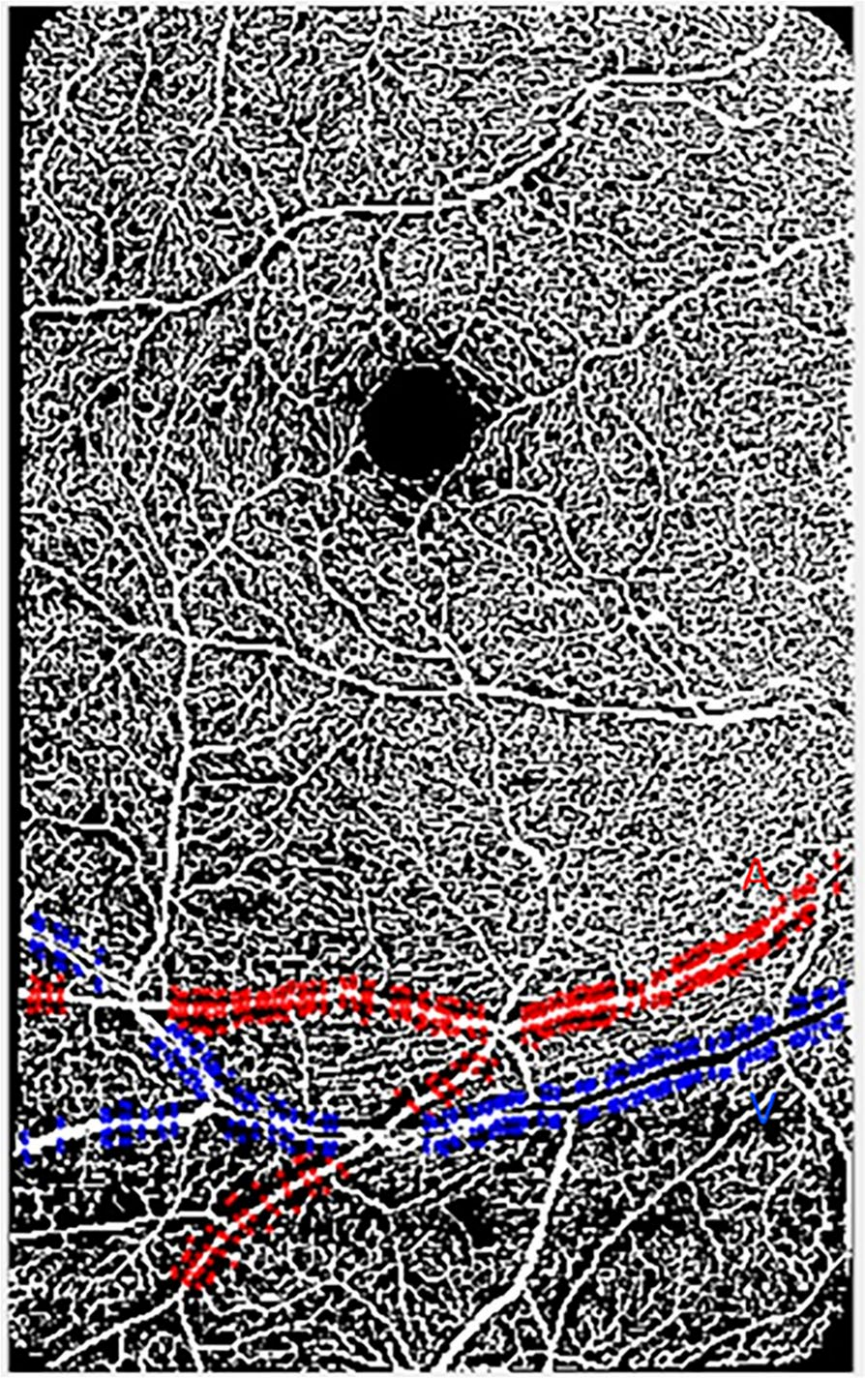
A vesselness filtered and Otsu thresholded montaged 20 × 20°OCTA image of the superficial vascular plexus in MATLAB of a 60-year-old cognitively unimpaired low-risk female. The image shows the central fovea (FAZ area = 0.48 mm^2^; FAZ effective diameter = 782 µm) and a paired arteriole (red “A”) and venule (blue “V”), 14.1° and 15.4°, respectively, inferior to the fovea. Evenly sampled points around the arteriole or venule showing the periarteriole (71.3 µm) and perivenule (58.5 µm) CFZs are shown. The arteriole diameter = 75.1 µm and the venule diameter = 124 µm. Arteriole linear distance of sampling for the periarteriole CFZ = 7.41 mm and venule linear distance of sampling for the perivenule CFZ = 6.67 mm

Raw OCTA images of the superficial vascular plexus (SVP) were exported from the HEYEX software as.tiff files (5.7 μm/pixel lateral resolution) into a custom programming software (MATLAB, Mathworks) where they were automatically cropped to eliminate the infrared component and include only the angiogram (Fig. 1a). The SVP was defined as the composite retinal vasculature from the inner limiting membrane to the inner plexiform layer/inner nuclear layer boundary [22, 23]. Images of the central foveal region and those of paired arterioles and venules inferior to the fovea were then automatically montaged using an image processing software (i2k Retina software) to generate a wider field of view of the paired vessels (Fig. 1a). The montaged images were then also exported into a custom programming software (MATLAB, Mathworks) for further processing. First, a vesselness filter was applied to the images [45, 46] to increase the probability of resolving a vessel at a specific location in the image when it is actually present versus noise or motion artifact (Fig. 1b). Next, Otsu thresholding method [47] was applied to the resultant image to reduce background noise (Fig. 1c) as have been done previously [23]. For all participants, we analyzed the mid-peripheral CFZs of the montaged images (Figs. 1 and 2) in an approximately linear pattern evenly along each sampled major arteriole or venule (first and second order branches). The average vessel distance from the fovea, vessel diameter, and vessel linear distance of sampling did not differ between the two groups, all *p* > 0.05 (Table 1).

**Table 1:** Covariates compared between low- and high-risk cognitively unimpaired older adults

Covariates	Low-risk cognitively unimpaired older adults (mean ± SD)	High-risk cognitively unimpaired older adults (mean ± SD)	*p*-value
Arteriole diameter	87.7 ± 12.1 µm	92.0 ± 14.5 µm	0.097
Venule diameter	113 ± 13.9 µm	116 ± 15.0 µm	0.29
Arteriole distance from the fovea	13.6 ± 2.38°	14.4 ± 2.33°	0.078
Venule distance from the fovea	14.9 ± 1.76°	15.2 ± 1.70°	0.47
Arteriole linear distance of sampling	6.39 ± 1.67 mm	6.29 ± 1.39 mm	0.73
Venule linear distance of sampling	6.05 ± 0.90 mm	5.88 ± 1.07 mm	0.37

**Fig. 3:**
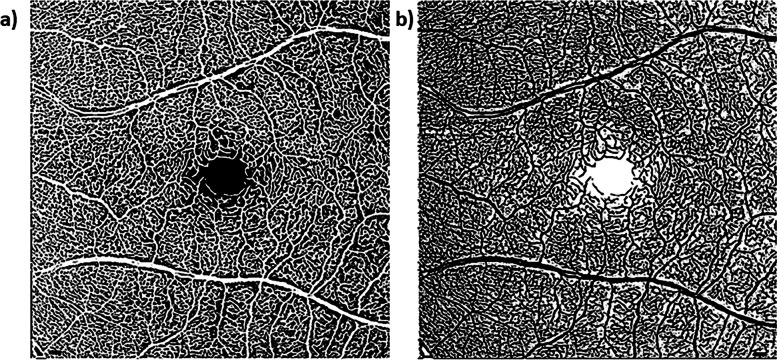
Computation of vessel density in a 20 × 20° macular-centered OCTA image of a 74-year-old cognitively unimpaired high-risk female. **a** Processed (cropped, vesselness filtered, and Otsu thresholded) image. **b** Corresponding reversed contrast/negative image. A customized MATLAB script was used to count the number of white pixels in both the original processed (**a**; designated as vessels) and the reversed contrast images (**b**; designated as the non-perfused background). The number of pixels was then converted to mm^2^ based on the micron‐to‐pixel ratio in the *x* and *y* directions, as computed from the fiducial marks acquired from the HEYEX software as done previously [22–24]. The area of white pixels in mm^2^ designated as vessels was then added to that of the background to compute the total area of the image (~ 36 mm^2^). The vessel density was then computed as the ratio of the area of white pixels designated as vessels (**a**) to the total area of the image. Scale bar: 200 µm

For vessel density computation, only the 20 × 20° macular-centered OCTA images were processed (cropped, vesselness filtered, and Otsu thresholded) for further image analysis (Fig. 3).

### Computation of Euclidean distances for mid-peripheral CFZs, vessel distance from fovea, vessel diameter, and vessel linear distance of sampling

Euclidean distances for the mid-peripheral CFZs, vessel distance from fovea, and vessel diameter were computed using similar formulas previously applied for CU young and older adults (Eqs. 1–3) [22, 23]. In this current paper, we also computed the linear distance of sampling (length of the vessel used for CFZ computation) for the mid-peripheral CFZs along the sampled arteriole or venule (Eq. 4). Briefly, we computed the mid-peripheral CFZ width in microns as the linear distance from the edge of an arteriole (periarteriole CFZ) or venule (perivenule CFZ) to the middle of the nearest capillary. The middle of the nearest capillary was used instead of the edge of the capillary because OCTA does not have enough lateral resolution to truly resolve the edge of a lumen of a capillary compared to other advanced retinal imaging modalities such as adaptive optics scanning laser ophthalmoscope (AOSLO). A custom MATLAB program automatically recorded to an Excel file the *x* and *y* coordinates from points evenly sampled perpendicular to an arteriole or venule and the middle of the nearest capillary (Fig. 2). The evenly sampled points in MATLAB included points perpendicular above and below a paired arteriole or venule as well as the center of the fovea.

Equations 1–4 were then used to compute the Euclidean distances for the mid-peripheral CFZs (periarteriole and perivenule CFZ), vessel distance from the fovea, vessel diameter, and vessel linear distance of sampling, respectively, using the *x* and *y* coordinates written by MATLAB into Excel. The outcome of each equation produced an Euclidean distance in pixels. For the mid-peripheral CFZ width (Eq. 1), vessel diameter (Eq. 3), and vessel linear distance of sampling (Eq. 4), the Euclidean distances in pixels were then converted into microns by multiplying them by the micron-to-pixel ratio in the *x* and *y* directions, as computed from the vendor software fiducial marks (Heidelberg Engineering, Table 1). For the vessel distance from the fovea (Eq. 2, Table 1), each Euclidean distance in microns was divided by 300 to convert them into degrees (assuming 300 µm =  ~ 1°).

1 $$\mathrm{Mid}-\mathrm{peripheral\,CFZ\,width}= {\left[{\left({\mathrm{X}}_{\mathrm{large\,vessel\,edge}}- {\mathrm{X}}_{\mathrm{capillary}}\right)}^{2}+ {\left({\mathrm{Y}}_{\mathrm{large\,vessel\,edge}}- {\mathrm{Y}}_{\mathrm{capillary}}\right)}^{2}\right]}^{\wedge 1/2}$$

where large vessel refers to a paired arteriole or venule, and capillary represents the middle of the nearest capillary (Fig. 2).

2 $$\mathrm{Vessel\,distance\,from\,fovea}= {\left[{\left({\mathrm{X}}_{\mathrm{vessel\,top}}- {\mathrm{X}}_{\mathrm{fovea}}\right)}^{2}+ {\left({\mathrm{Y}}_{\mathrm{vessel\, top}}- {\mathrm{Y}}_{\mathrm{fovea}}\right)}^{2}\right]}^{\wedge 1/2}$$

where vessel top refers to the top of a paired arteriole or venule, and fovea represents the middle of the FAZ (Fig. 2).

3 $$\mathrm{Vessel\,diameter}= {\left[{\left({\mathrm{X}}_{\mathrm{vessel \,top}}- {\mathrm{X}}_{\mathrm{vessel \,bottom}}\right)}^{2}+ {\left({\mathrm{Y}}_{\mathrm{vessel \,top}}- {\mathrm{Y}}_{\mathrm{vessel \,bottom}}\right)}^{2}\right]}^{\wedge 1/2}$$

where vessel top/bottom refers to the top/bottom of a paired arteriole or venule (Fig. 2).

4 $$\mathrm{Vessel\,linear\,distance\,of\,sampling}: {\sum \left[{\left({X}_{vessel \,top (n)}- {X}_{vessel \,top (n+1)}\right)}^{2}+ {\left({Y}_{vessel\, top (n)}- {Y}_{vessel \,top (n+1)}\right)}^{2}\right]}^{\wedge 1/2}$$

where vessel top refers to the top of a paired arteriole or venule, and *n* refers to a series of evenly sampled neighboring coordinates (Fig. 2).

### Computation of FAZ size and FAZ effective diameter

The area-finding tool (lasso tool) of the vendor software was used to delineate and compute the FAZ area in mm^2^. FAZ effective diameter was then computed from the FAZ area values. The FAZ effective diameter in microns was defined as the diameter of a circle whose area was equivalent to the known FAZ areas; FAZ effective diameter = (4*FAZ area/*π*)^1/2^, similar to that done for the CU young and older adults [22, 23].

### Computation of vessel density

Vessel density computation has been previously described [48]. Briefly, after the macular-centered 20 × 20° images have been processed (cropped, vesselness filtered, and Otsu thresholded; Fig. 3a) as described above, reversed contrast/negatives of those images were created (Fig. 3b). A customized MATLAB script was then used to count the number of white pixels in both the original processed (Fig. 3a; designated as vessels) and the reversed contrast images (Fig. 3b; designated as the non-perfused background). The number of pixels was then converted to mm^2^ based on the micron‐to‐pixel ratio in the *x* and *y* directions, as computed from the fiducial marks acquired from the HEYEX software as done previously [22–24]. The area of white pixels in mm^2^ designated as vessels was then added to that of the background to compute the total area of the image which was expected to be ~ 36 mm^2^. The vessel density was then computed as the ratio of the area of white pixels designated as vessels (Fig. 3a) to the total area of the image.

### Statistical analyses

All statistical analyses were completed using statistical software (IBM SPSS Statistics for Windows, version 29; IBM Corp., Armonk, NY, USA). All values were descriptively presented as mean ± SD. Considering a sample size of 107 eyes with kurtosis and skewness of ≤  ± 3.50 for all our outcome variables, normality was assumed, and parametric tests were performed for the data analyses. We previously found the perivenule CFZ to be significantly positively associated with vessel distance from the fovea and vessel diameter in CU young adults [22]. In this current study, vessel distance from the fovea, vessel diameter, and vessel linear distance of sampling served as covariates when comparing the periarteriole and perivenule CFZ between the two groups of participants (Table 1). An independent sample *t* test was performed to compare the covariates (Table 1) between the two groups. Since the covariates did not significantly differ between the two groups (Table 1), the periarteriole and perivenule CFZ width was compared between the high-risk and low-risk participants using an independent sample *t* test. A similar independent sample *t* test was used to compare the FAZ size, FAZ effective diameter, and vessel density between the two groups. Cohen’s *d* was used to measure the effect size. A paired sample *t* test was used to compare the periarteriole versus perivenule CFZ in both the low-risk and high-risk CU older adults. A logistic regression model combining the periarteriole and perivenule CFZ (mid-peripheral CFZs) was initially used to provide predictive/probability values. A receiver operating characteristic (ROC) model with these predictive/probability values was then used to assess the sensitivity, specificity, and area under the curve (AUC) to distinguish between high-risk and low-risk participants. Another logistic regression model combining the mid-peripheral CFZs, FAZ effective diameter, and vessel density was also utilized to provide predictive/probability values. FAZ effective diameter was chosen instead of FAZ size because we found the former to be less variable than the latter in our previous study [23]. A second ROC model with these predictive/probability values assessed the sensitivity, specificity, and AUC to distinguish between high-risk and low-risk participants. In both ROC scenarios, a Youden’s index that maximizes sensitivity and moderates specificity was chosen to create cutoffs. Such a Youden’s index was chosen because the goal of our study is to develop a screening test, and hence, such a test/model should have good sensitivity even if it has moderate specificity. The following AUC classification was used for our study; 0.5–0.6 = unsatisfactory, 0.6–0.7 = satisfactory, 0.7–0.8 = good, 0.8–0.9 = very good, and 0.9–1 = excellent. A *p*-value of < 0.05 was considered statistically significant.

## Results

### Characteristics of the mid-peripheral CFZs in low-risk and high-risk CU older adults

**Fig. 4:**
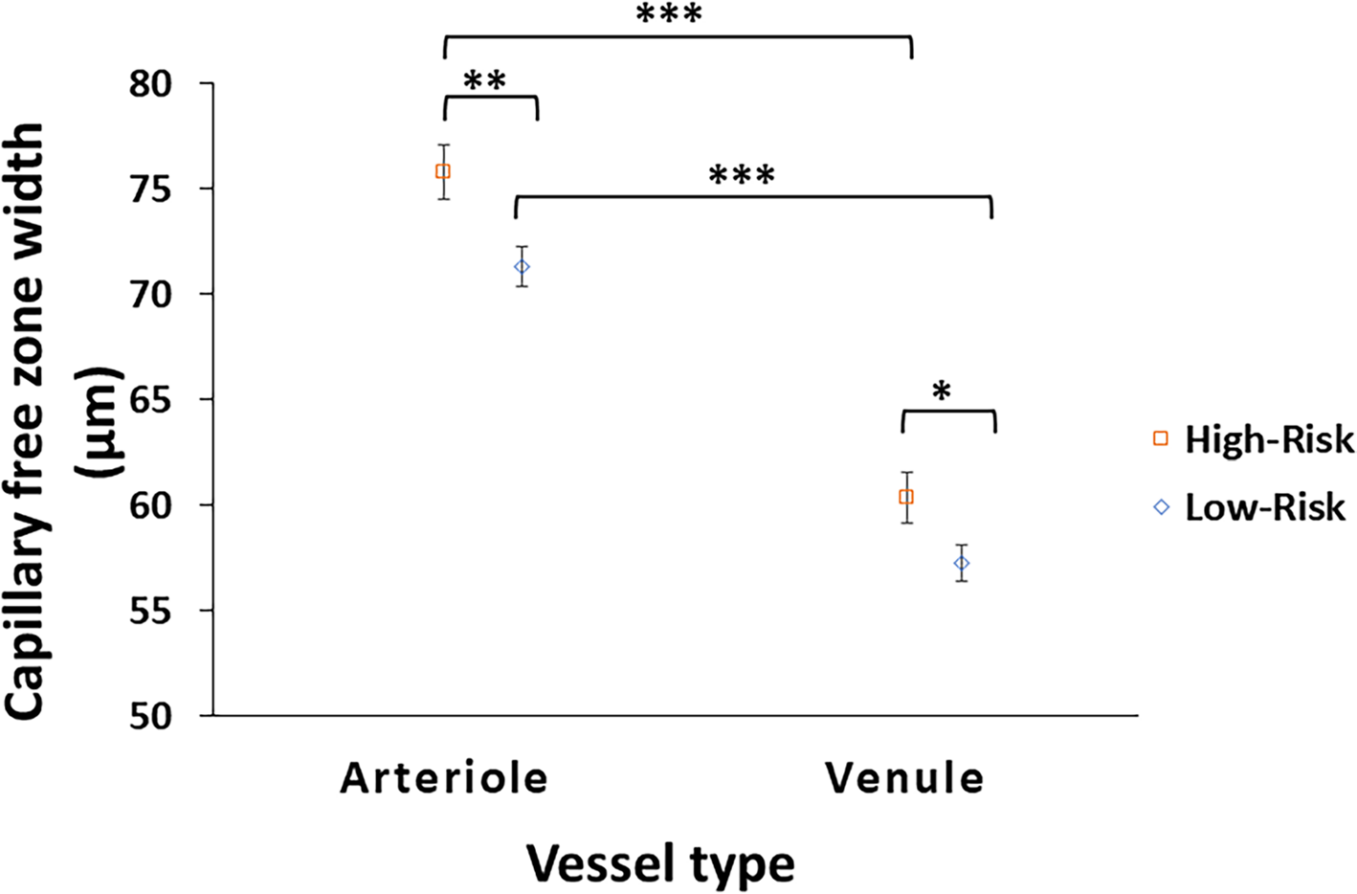
The mean mid-peripheral CFZ width with standard error of the mean error bars for arterioles and venules in cognitively unimpaired (CU) low- and high-risk older adults. The periarteriole CFZ width is significantly greater than the perivenule CFZ width in both groups (*p* < 0.001) similar to that reported previously [22, 23]. The periarteriole CFZ width of the CU high-risk older adults is significantly greater than that of the CU low-risk older adults, *p* = 0.005, with a medium effect size (Cohen’s *d*) = 0.55. Similarly, the perivenule CFZ width of the CU high-risk older adults is also significantly greater than that of the CU low-risk older adults, *p* = 0.034, with a small effect size (Cohens’ *d*) = 0.42. *p < 0.05, **p < 0.01, ***p < 0.001

In low-risk CU older adults, the periarteriole CFZ width (71.3 ± 7.07 µm; range = 58.1–92.1 µm; 95% CI for mean = 69.5–73.2 µm; SEM = 0.94) was significantly larger than the perivenule CFZ width (57.3 ± 6.40 µm; range = 47.4–86.3 µm; 95% CI for mean = 55.6–59.0 µm; SEM = 0.85), *t*(56) = 13.1, *p* < 0.001 (Fig. 4). Similarly in high-risk CU older adults, the periarteriole CFZ width (75.8 ± 9.19 µm; range = 60.0–111 µm; 95% CI for mean = 73.2–78.4 µm; SEM = 1.30) was significantly larger than the perivenule CFZ width (60.4 ± 8.55 µm; range = 48.2–93.2 µm; 95% CI for mean = 58.0–62.8 µm; SEM = 1.21), *t*(49) = 13.3, *p* < 0.001 (Fig. 4).

### Comparison of the mid-peripheral CFZ width between low-risk and high-risk CU older adults

**Fig. 5:**
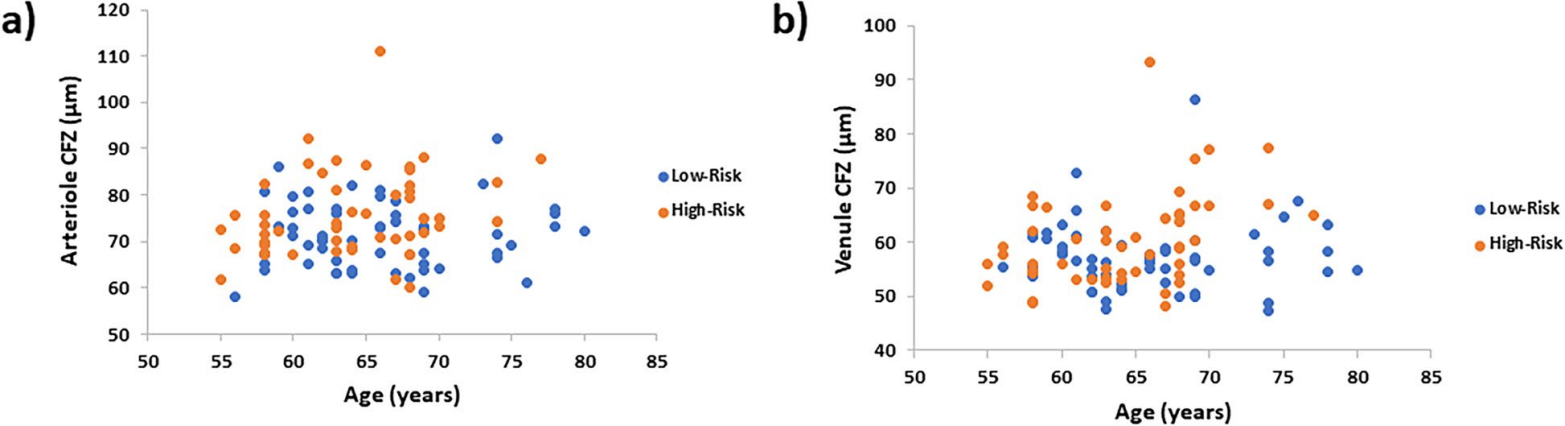
Scatter plot showing the individual variations in the mid-peripheral CFZs in low- and high-risk cognitively unimpaired (CU) older adults. **a** Scatter plot of the periarteriole CFZ width versus age showing individual variations in the periarteriole CFZs in low- and high-risk CU older adults. A trend towards large periarteriole CFZs can be observed for the high-risk CU older adults. **b** Scatter plot of perivenule CFZ width versus age showing individual variations in the perivenule CFZs in low- and high-risk CU older adults. A trend towards large perivenule CFZs can be observed for the high-risk CU older adults

**Fig. 6:**
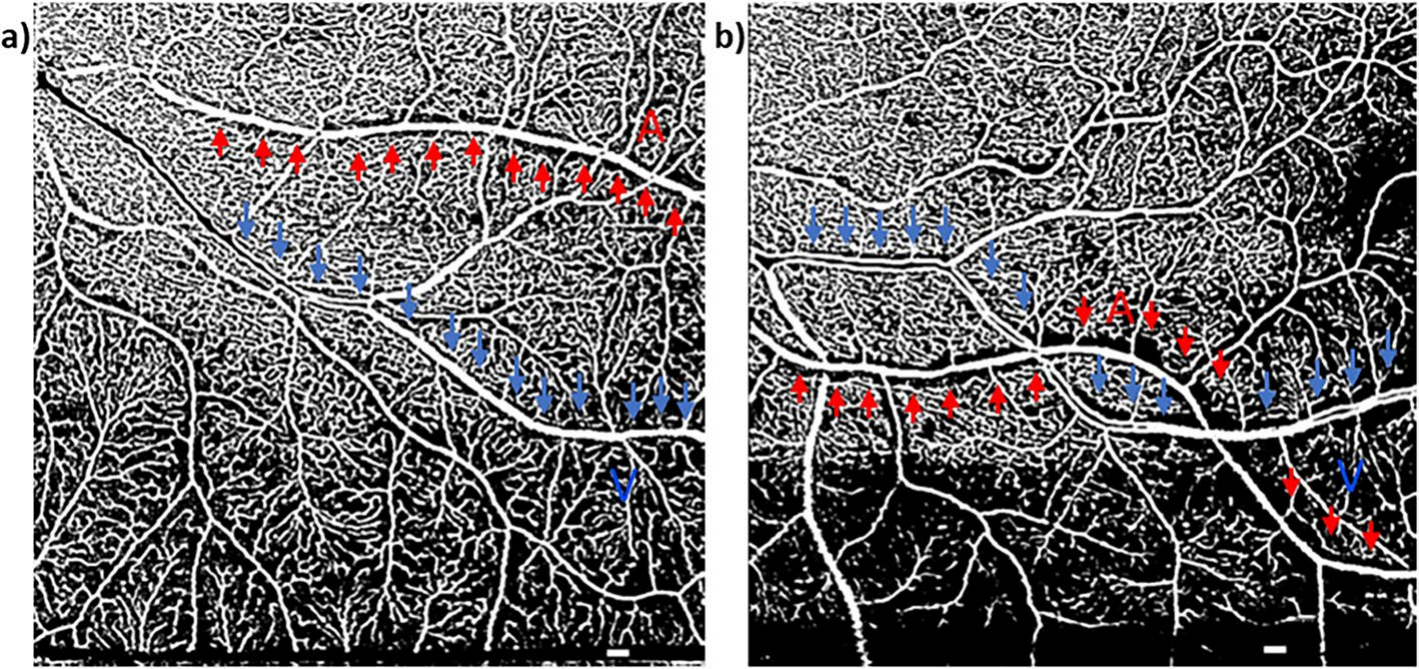
Processed OCTA images in MATLAB showing larger periarteriole (111 µm; vessel linear distance = 4.2 mm) and perivenule CFZ (93.2 µm; vessel linear distance = 6.7 mm) in a 66-year-old high-risk cognitively unimpaired (CU) female (**b**) than those observed in a 68-year-old low-risk CU female (**a**; periarteriole CFZ = 72.6 µm, vessel linear distance = 4.7 mm; perivenule CFZ = 64.9 µm, vessel linear distance = 5 mm). Periarteriole CFZ; red “A” and arrows. Perivenule CFZ; blue “V” and arrows. Scale bar: 200 µm

The mean periarteriole CFZ width of the high-risk CU older adults (75.8 ± 9.19 µm) was significantly larger than that of the low-risk CU older adults (71.3 ± 7.07 µm), *t*(105) =  − 2.85, *p* = 0.005, with a medium effect size (Cohen’s *d*) = 0.55 (Figs. 4, 5, and 6). The mean periarteriole CFZ width (75.8 µm) of the high-risk CU older adults was outside the 95% CI of the low-risk CU adults (69.5–73.2 µm). There was no overlap between the 95% CI of the mean periarteriole CFZ width of the high-risk group (73.2–78.4 µm) and that of the low-risk group (69.5–73.2 µm). Similarly, the mean perivenule CFZ width of the high-risk CU older adults (60.4 ± 8.55 µm) was significantly greater than that of the low-risk CU older adults (57.3 ± 6.40 µm), *t*(105) =  − 2.15, *p* = 0.034, with a small effect size (Cohens’ *d*) = 0.42 (Figs. 4, 5, and 6). Also, the mean perivenule CFZ width of the high-risk CU older adults (60.4 µm) was outside the 95% CI of the low-risk CU (55.6–59.0 µm). However, there was an overlap between the 95% CI of the mean perivenule CFZ width of the high-risk group (58.0–62.8 µm) and that of the low-risk group (55.6–59.0 µm).

Processed OCTA images in MATLAB are shown in Fig. 6 to demonstrate an example of large mid-peripheral CFZs in a high-risk CU older adult versus a low-risk CU older adult.

### Comparison of FAZ parameters and vessel density between low-risk and high-risk CU older adults

The FAZ area of the high-risk CU older adults (0.345 ± 0.142 mm^2^) did not significantly differ from that of the low-risk CU older adults (0.361 ± 0.115 mm^2^), *t*(105) = 0.64, *p* = 0.52. Similarly, the FAZ effective diameter for the high-risk CU older adults (648 ± 139 µm) did not significantly differ from the low-risk CU older adults (669 ± 110 µm), *t*(105) = 0.86, *p* = 0.39. Vessel density also did not differ between the high-risk group (0.507 ± 0.039) and the low-risk group (0.497 ± 0.041), t(105) =  − 1.27, *p* = 0.21.

### ROC model of mid-peripheral CFZs to distinguish between low-risk and high-risk CU older adults

**Fig. 7:**
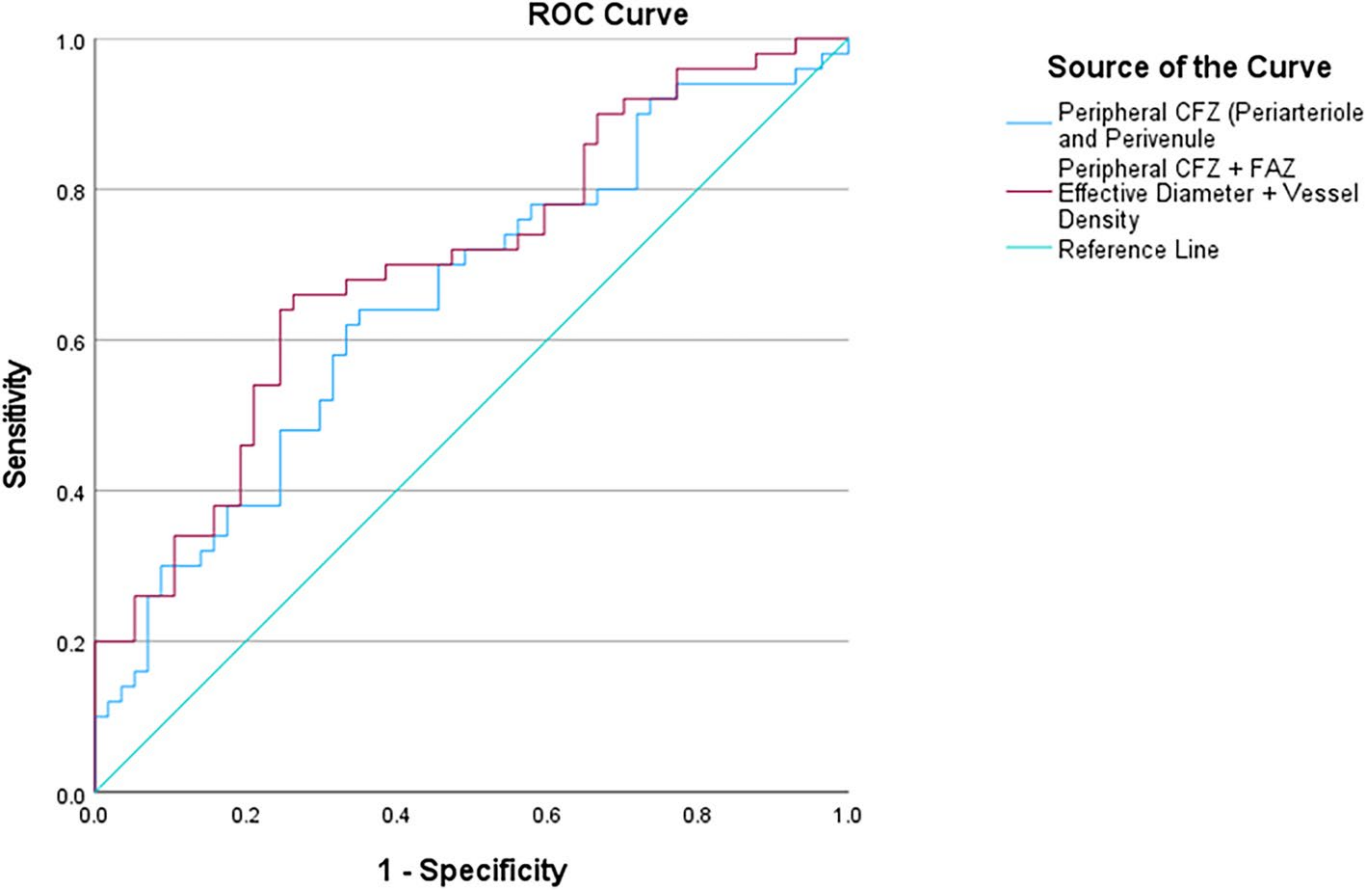
A receiver operating characteristic (ROC) curve showing a model of the mid-peripheral CFZ (periarteriole and perivenule CFZ) and multimodal model of the mid-peripheral CFZ, FAZ effective diameter, and vessel density to distinguish between low- and high-risk cognitively unimpaired older adults. The ROC model combining the periarteriole and perivenule CFZ width (mid-peripheral CFZs) is statistically significant, AUC = 0.65 (95% CI = 0.55–0.76), *p* = 0.006. Interestingly, the multimodal model which combined the mid-peripheral CFZs, FAZ effective diameter, and vessel density yielded a better model which is also statistically significant, AUC = 0.70 (95% CI = 0.60–0.80), *p* < 0.001

The ROC model combining the periarteriole and perivenule CFZ width (mid-peripheral CFZs) was statistically significant with an AUC = 0.65 (95% CI = 0.55–0.76), *p* = 0.006 (Fig. 7). A Youden’s index of 0.244 (which maximizes sensitivity and moderates specificity) was chosen to create a cutoff predictive value of 0.423. The cutoff predictive value corresponded to a periarteriole CFZ width of ≥ 71.2 µm and perivenule CFZ width of ≥ 56.8 µm for the high-risk positive state with a sensitivity of 70% and a specificity of 54%.

### ROC model of mid-peripheral CFZs, FAZ effective diameter, and vessel density to distinguish between low-risk and high-risk CU older adults

Interestingly, an ROC model which combined the mid-peripheral CFZs, FAZ effective diameter, and vessel density yielded a better model which was also statistically significant with an AUC = 0.70 (95% CI = 0.60–0.80), *p* < 0.001 (Fig. 7). A Youden’s index of 0.314 was chosen to create a cutoff predictive value of 0.443. The cutoff predictive value corresponded to a periarteriole CFZ width of ≥ 72.2 µm, perivenule CFZ width of ≥ 66.6 µm, FAZ effective diameter of ≥ 731 µm (FAZ size of ≥ 0.42 mm^2^), and vessel density of ≥ 0.47 for the high-risk positive state with a sensitivity of 70% and a specificity of 61%.

## Discussion

In our current study of high-risk for AD defined by the presence of at least one APOE e4 allele and a first-degree family history of AD, we found statistically significant larger periarteriole and perivenule CFZs (mid-peripheral CFZs) in the high-risk CU older adults compared to the low-risk CU older adults. FAZ and vessel density metrics did not significantly differ between these two groups. The moderate effect size for the periarteriole CFZ shows that it has better potential to serve as a future clinical biomarker than the small effect size we found for the perivenule CFZ. A statistically significant satisfactory ROC model including the mid-peripheral CFZs distinguished between the low- and high-risk CU older adults, which was modestly better with increased specificity when a multimodal ROC model combined the mid-peripheral CFZs with other retinal vascular metrics (FAZ effective diameter and vessel density) for a good AUC to distinguish between the two groups. Our data provide cutoff predictive values for periarteriole and perivenule CFZ widths for the high-risk positive state in this multimodal model to yield a sensitivity of 70% and a specificity of 61%.

The inner retinal blood barrier essentially controls nutrient flow to the neural retinal; specifically, the inner retinal neurons [49]. A mid-peripheral CFZ represents the distance that oxygen, and nutrients must diffuse to reach the neural retina, with larger distances indicating potential ischemia [22, 23]. In addition, the mid-peripheral CFZs have an anatomical resemblance to the perivascular spaces seen in the brain parenchyma (Virchow-Robin spaces). These spaces play an important role in nutrient distribution and may be a key element of the recently described glymphatic pathway; a network of perivascular spaces involved in the removal of cerebral solutes and cell byproducts such as beta-amyloid (Aβ) and tau [50–52]. In AD, breakdown of the inner retinal blood barrier, pericyte loss, and capillary non-perfusion or dropout occur [36, 37] leading to potential enlargement of the mid-peripheral CFZs around arterioles and venules in the retina. Also, there is dilatation of the cerebral perivascular spaces (a postulated indirect neuroimaging biomarker of impaired glymphatic function) in AD patients as shown by magnetic resonance imaging (MRI) [50–52], indicating possible changes in the perivascular spaces in the retina (mid-peripheral CFZs) of these patients. APOE e4 genotype has been associated with vascular impairment in AD, and it is an important risk marker for abnormal Aβ accumulation and impaired clearance within the brain vasculature [53–56], positing similar changes in the retinal vasculature of these patients. Building on these prior studies and in support of our hypothesis, we found evidence that the mid-peripheral CFZs were significantly enlarged in CU older participants at high risk for AD compared to age-matched low-risk CU older participants, which posits similar perturbation in the retinal vasculature as has been reported in the brain [50–56]. Larger retinal mid-peripheral CFZs in the high-risk group indicates large spaces or passageways around the retinal arterioles and venules in these patients for diffusion of oxygen, nutrients, and other waste products of metabolism between the retinal vascular and neural system.

Perivascular spaces in the brain basically include gaps or passageways around arterioles, capillaries, and venules along which a range of substances can move [50–52] similar to the structure and function of the retinal mid-peripheral CFZs (retinal perivascular spaces) [22, 23]. However, it is currently under debate whether MRI-visible perivascular spaces surround both arterioles and venules [57–59]. Most MRI at conventional strengths cannot easily distinguish between perforating arterioles and venules [50]. The use of 7-T MRI has demonstrated that MRI-visible perivascular spaces are spatially more correlated with arterioles but not venules [60]. The use of lower field T2 sequence (if images are of good enough quality) to visualize perivascular spaces and venules in the centrum semiovale [50, 61] suggests that perivascular spaces are distinct from venules [50, 62]. Thus, most evidence in the literature suggests that MRI-visible perivascular spaces are periarteriolar rather than perivenular [60–62]. Since the human retina is an extension of the brain, and perivascular spaces in the brain are more periarteriolar than perivenular [60–62], this may explain the moderate effect size for the periarteriole CFZ compared to the small effect size for the perivenule CFZ.

We did not find significant differences between the two AD risk groups with respect to FAZ size, FAZ effective diameter, and vessel density. The lack of significant differences in the FAZ metrics in our study is similar to that reported in previous studies that compared the FAZ metrics between participants with MCI and controls [29, 31], as well as preclinical AD and controls [30, 32]. The lack of significant differences in the FAZ metrics between the two groups in our study could be explained by the large individual variability in the FAZ metrics even in the CU older adult population [23, 25–28] leading to the overlaps between the two groups. Interestingly, a paradoxical smaller FAZ size has been reported in participants with genetic risk for AD (APOE e4) compared to those without in a previous study [15]. The lack of significant differences in vessel density between the two groups is also similar to that reported in previous studies that compared vessel density metrics between controls and preclinical AD [32], as well as MCI and controls [29, 31]. Even in studies that found significant differences, these differences were found in the later stages of the disease (AD vs. MCI, and AD vs. controls) rather than in the early stages [31, 33]. A longitudinal study reported reduced baseline retinal vessel density metrics in APOE e4 carriers compared to non-carriers, but these metrics were not significantly different between the two groups after a 2-year follow-up [16]. Interestingly, one study found a paradoxical large vessel density in preclinical AD patients compared to controls [30]. The lack of significant differences in vessel density between the two groups could be explained by the fact that OCTA vessel density computations are influenced by noise in the image along with variable anatomic features such as vessel diameter [20, 21, 23].

A single retinal biomarker related to either neural (retinal nerve fiber layer; RNFL thickness), vascular (vessel density and FAZ metrics), or proteinopathy changes (retinal amyloid/inclusion bodies) may not be sensitive and specific to AD. For example, RNFL thickness that has been shown to be thinner in AD [63–65] is also implicated in glaucoma [66–68]. Thus, it becomes possible that a retinal biomarker study for AD could include participants with early glaucoma who have not yet been formally diagnosed with glaucoma. Retinal vascular metrics, such as vessel density and FAZ size, that have been previously studied in AD [29–31, 33, 34] are also implicated in diabetic retinopathy [69–71]. Changes in these retinal vascular metrics precede clinically detected diabetic retinopathy (diagnosed using dilated fundus examination or color fundus images) [69–71]. Thus, an AD retinal biomarker study that includes diabetics but has excluded clinical diabetic retinopathy may have participants with changes in retinal vessel density and FAZ size that are unrelated to AD. Also, retinal amyloid/inclusion bodies indicated in AD [72, 73] are also found in retinal drusenoid structures in age-related macular degeneration (AMD) [74]. It must however be noted that other studies have found no association between a family history of AD/APOE e4 genotype and the presence of drusen, and that amyloid deposits are distinct from drusenoid structures [72, 75]. Without a multimodal imaging model of blue autofluorescence imaging, color fundus, and spectral domain OCT, drusenoid structures in AMD may be falsely counted as retinal amyloid or inclusion bodies. Thus, several possible retinal biomarkers that have been investigated for AD are also affected by other retinal disease processes. While a retinal vascular metric, e.g., the mid-peripheral CFZs may have advantages over other known vascular metrics (FAZ and vessel density metrics), it currently appears that the way forward as a field is to utilize a multimodal approach that combines all the vascular metrics to improve sensitivity and specificity of these metrics for early AD risk detection. This proposal is supported by our finding of a multimodal ROC model that combined the mid-peripheral CFZs with other retinal vascular metrics to yield a better ROC model to distinguish between two CU groups with different risks of AD, as well as by a previous study that investigated a multimodal model of different types of fractal and lacunarity analysis to distinguish between cognitively impaired and CU older adults [76].

Given the universality of blood collection in medical settings, blood-based biomarkers have the potential to improve widespread access to AD screening and diagnosis in both high- and low-resource areas. These blood-based biomarkers for AD include Aβ42/Aβ40 ratio [10], p-tau 181 [11], p-tau 217 [77], p-tau 231 [78], neurofilament light protein (NfL) [12], and glial fibrillary acidic protein (GFAP) [13]. p-tau 217 shows better results at detecting AD pathology (including preclinical AD) and for monitoring of disease progression [77, 79, 80]. However, the robustness of the measured Aβ42/Aβ40 ratio in blood plasma is only 0.9-fold times lower in patients with brain amyloidosis compared to controls and therefore the challenge with implementing blood-based Aβ42/Aβ40 ratio for screening purposes is the smaller effect size when compared to its CSF counterparts [81]. This could be explained by the contamination of results based on the fact that plasma Aβ is derived from peripheral sources (downstream effect) [82, 83] unlike the retina, which is a direct extension of the brain, and can also be influenced by genetic factors and renal function [82]. With respect to p-tau biomarkers, the robustness ranges from low to high effect size from preclinical AD to prodromal AD, respectively, with the highest levels in AD dementia with p-tau 181 and also p-tau 231 both not performing as well as p-tau 217 [79, 80], positing that blood-based biomarkers may do better in later rather than early disease. This could be explained by the fact that unlike the retina which is a direct extension of the brain and can detect subtle early changes in AD, blood-based biomarkers are derived via a downstream effect from the CNS. While retinal biomarkers may have some advantages over blood-based biomarkers and vice-versa, the argument today cannot be choosing one over the other, but rather investigating how both groups of biomarkers are related to each other, as well as developing a model that incorporates both biomarkers to improve the sensitivity, specificity, and AUC for early detection of AD. The next phase of our research will investigate the relationship between the mid-peripheral CFZs (especially the periarteriole CFZ) and the abovementioned blood-based biomarkers as well as a model that incorporates both biomarkers.

Our other future research endeavors will investigate the associations between the mid-peripheral CFZ width (retinal perivascular spaces; especially the periarteriole CFZ width) [22, 23] and quantitative features (length, width, volume, etc.) of MRI-defined perivascular spaces in the centrum semiovale in AD [50–52]. Our results show that the mid-peripheral CFZs are enlarged in CU older adults at high risk for AD compared to CU low-risk participants. Also, in AD, there is impairment in the drainage of fluid in the brain glymphatic system which leads to the accumulation of Aβ and dilatation of the perivascular spaces [50–52]. From a mechanistic perspective, the goal will be to use mid-peripheral measures of variability in retinal vasculature (mid-peripheral CFZs) as an intriguing approach to test hypotheses about potential vascular contributions to AD.

An inherent limitation of our current study is the limits of lateral resolution of OCTA technology. However, OCTA has better axial resolution than other superior lateral resolution devices, such as AOSLO. There are new developments in the technology to improve the field of view, and speed of image acquisition, which may be valuable to improve image quality and analysis in future studies. The cross-sectional nature of this proof-of-concept study provides support for a future longitudinal study to investigate the within and between subject changes over time (especially for the periarteriole CFZs) and as well as the association with blood-based and brain AD biomarkers (PET Aβ and tau SUVR). To ensure clinical applicability of the mid-peripheral CFZs (especially the periarteriole CFZs) for early AD risk detection, the MATLAB scripts used for their computations will need to be commercialized and incorporated into the current OCTA imaging modalities in the clinic in future research. At the present time, there is little broad agreement on how best to compute vessel density from OCTA images, as processing technology is still relatively new [48]. There is a rapidly growing number of published methods for OCTA signal analyses and data reporting, and currently little agreement on standard metrics [48]. In our current study, we used our previously reported methodology for vessel density computation for our OCTA images [48].

## Conclusions

In our current study, we found significantly larger periarteriole and perivenule CFZs (mid-peripheral CFZs) in high-risk CU older adults compared to similarly aged low-risk CU older adults. In terms of clinical relevance, the periarteriole CFZs had a better effect size than the perivenule CFZs indicating the former has a better potential for clinical applicability for early AD risk detection compared to the latter. A multimodal ROC model that combined the mid-peripheral CFZs with other retinal vascular metrics (including the FAZ effective diameter and vessel density) yielded a better ROC model to distinguish between the two groups of participants compared to the mid-peripheral CFZs alone. This finding indicates that a multimodal retinal vascular approach would be more valuable for early AD risk detection to utilize in future research going forward.

## Data Availability

The datasets used and/or analyzed during the current study are available from the corresponding author upon reasonable request.

## Abbreviations

AD: Alzheimer’s disease
CU: Cognitively unimpaired
CFZs: Capillary free zones
MoCA: Montreal Cognitive Assessment
FAZ: Foveal avascular zone
PET: Positron emission tomography
CSF: Cerebrospinal fluid
MCI: Mild cognitive impairment
RGC: Retinal ganglion cells
OCTA: Optical coherence tomography angiography
AOSLO: Adaptive optics scanning laser ophthalmoscope
APOE: Apolipoprotein E
RBANS-U: Repeatable Battery for the Assessment of Neuropsychological Status Update
DMI: Delayed Memory Index
ARIAS: Atlas of Retinal Imaging in Alzheimer’s Study
DNA: Deoxyribonucleic acid
PCR: Polymerase chain reaction
SVP: Superficial vascular plexus
AUC: Area under the curve
ROC: Receiver operating characteristic
NfL: Neurofilament light
GFAP: Glial fibrillary acidic protein
AMD: Age-related macular degeneration
CNS: Central nervous system
Aβ: Amyloid beta
SUVR: Standardized uptake value ratio
SEM: Standard error of the mean
